# Comparative assessment of commercial ELISA kits for detection of HIV in India

**DOI:** 10.1186/1756-0500-7-436

**Published:** 2014-07-07

**Authors:** Srijita Nandi, Susmita Maity, Somesh Chandra Bhunia, Malay Kumar Saha

**Affiliations:** 1 National HIV Reference Laboratory, National Institute of Cholera and Enteric Diseases, Beliaghata, 700010 Kolkata, India

**Keywords:** HIV, ELISA, Sensitivity, Specificity, Efficiency, Sera panel, Seroconversion panel

## Abstract

**Background:**

India harbors the 3^rd^ highest HIV infected population globally. The magnitude of the HIV detection challenge is enormous. ELISA is the most commonly used screening technique for HIV. There is always an acute need for good quality ELISA kits. However, the quality evaluation data on Indian kits are very limited in comparison with internationally recognized kits. This study aimed to evaluate the performance and diagnostic usefulness of five commercially available ELISA kits which are frequently used in India.

**Findings:**

The ELISA kits evaluated using an in-house well characterized 100 member sera panel revealed 100% sensitivity for all the batches. However, batch to batch variation in terms of specificity, positive predictive value (PPV) and efficiency, although not statistically significant (p > 0.05), was observed. For specificity, the 3^rd^ generation kits (mean 99.6% to 99.3%) were comparatively better than the 4^th^ generation assays (97.2% to 96.9%). But the 4^th^ generation kits performed far better in the ability for early detection post HIV infection in the 25 member commercial seroconversion panel with a margin of at least 22 days and as high as 35 days than the 3^rd^ generation assays.

**Conclusions:**

The commercial ELISA kits with 100% sensitivity seem appropriate for HIV screening. The ability of early detection post HIV infection favors use of 4^th^ generation kits for ensuring HIV free blood for transfusion. Lot to lot variations, especially kits having the specificity level ≤98.0%, indicate the need for a regular mechanism of kit evaluation for each batch for procuring kits appropriate for intended use.

## Findings

### Background

HIV is a major global public health issue [1]. For assuring a safe blood supply and preventing HIV infection, proper and accurate detection of HIV is essential [2]. In India, diagnosis of HIV infection is a major challenge [3,4]. Several commercial assays are available for detection of HIV infection. ELISA is the most commonly used screening assay for HIV [2,5]. A number of ELISA kits for HIV detection with different principles are available. Nowadays, in India 3^rd^ generation ELISA are most commonly used. The 4^th^ generation assays are based on combined detection of antigen and antibodies simultaneously and reduce the diagnostic window period further, compared to third generation ELISA which is based on anti-HIV antibody assay [6-8]. The improved sensitivity for ELISA is mostly accompanied by a decreased specificity. In an Indian perspective, limited articles on evaluation and performance of ELISA kits are available [9] though HIV testing is being done for a vast numbers of individuals as well as large number of specimens for ensuring HIV free safe blood for transfusion. Being the 2^nd^ most populous country with the 3^rd^ largest burden of HIV in the world [10], the magnitude of HIV testing challenge in India is enormous and the appropriate response to the challenge is to ensure the quality of the assay kits suitable for the intended use. This study aims to evaluate the quality of commonly available commercial ELISA kits for their ability to detect HIV suitable for appropriate use in India.

### Materials and methods

The study was carried out at a National HIV Reference Laboratory designated for evaluation of diagnostic kits, including ELISA, in India. A well characterized, 100 members, in-house HIV serum panel was used to evaluate and compare the performance of the kits. The sera used for preparing the in-house panel were obtained anonymously from attendees of the Counseling and Testing Centre by taking informed consent as per the protocol approved by Institutional Ethical Committee of National Institute of Cholera and Enteric Diseases. Beside the negative and positive sera, this serum panel also contained low positive sera that have shown uniform results in all assays used for validation. The characterization of in-house panel was done using United States Food and Drug Administration (U.S. FDA) or Indian Central Drug Standard Control Organization (CDSCO) approved kits (Table 1). Samples non-reactive for all assays were defined as negative and reactive for all assays were defined as positive member in the panel. A commercial seroconversion panel (Lot# RP-018, Bio-Rad Laboratories, U.S.A) used to evaluate kits consists of a series of 25 specimens collected from an individual infected with HIV undergoing seroconversion.

**Table 1:** Details of kits used for characterization of in-house panel sera

**Source of sample**	**Name of panel**	**Test details**			
		**ELISA 1**	**ELISA 2**	**Rapid test**	**Confirmatory test**
ICTC	HIV Sera Panel (In-house)	Genetic Systems	Genedia HIV Ag-Ab ELISA	Rapid 1: Determine HIV1/2	Recombinant Immunoblot Assay
		HIV-1/HIV-2	Green Cross Life Science Corp. Korea	Inverness Medical Japan Co. Ltd. Japan	Chiron RIBA HIV-1/HIV-2 SIA
		Plus O EIA.			
		Bio-Rad Laboratories. USA	Reactivity range (pos specimens): 3.503-8.899	Rapid 2: HIV TRI-DOT + Ag	Ortho Clinical
					Diagnostics Ltd, USA
				J. Mitra & Co. Pvt. Ltd. India	AMPLICOR HIV-1 DNA Test
		Reactivity range			
					Version 1.5
					Roche Molecular Systems Inc. USA
		(pos specimens): 3.485-10.017			

Five batches each of 5 commonly available commercial ELISA kits, including 3^rd^ and 4^th^ generations, for HIV detection were evaluated (Table 2). All the kits were tested and results were validated strictly adhering to manufacturers’ instruction. The evaluation process maintained an unbiased method following a double blind procedure by using different personnel for pre-analytical and analytical testing sections. The final analysis of results with interpretation was done by the laboratory in-charge. The status of samples was unknown to the persons involved in pre-analytical and analytical procedures. The performance of kits was evaluated and compared in terms of sensitivity ([TP/(TP + FN)]×100), specificity ([TN/(TN + FP)]×100), positive predictive value (PPV = [TP/(TP + FP)]×100), negative predictive value (NPV = [TN/(TN + FN)]×100) and efficiency ([(TP + TN)/(TP + FN + TN + FP)]×100), where TP = number of true positives, TN = number of true negatives, FP = number of false positives and FN = number of false negatives [11]. Confidence Interval (CI) was used to address precision of the proportion estimates and the degree of confidence was set to 95% [12]. Chi-square analysis was performed to assess the variation for specificity, PPV and efficiency among different kits as well as in different batches of same kit.

**Table 2:** Performance of HIV ELISA kits with In-house Sera Panel

**Total no. of samples**	**Total no. of lots evaluated**		**Kit performance**									
**100**	**5 Lots**		**J. Mitra & Co. Pvt. Ltd.**		**Span Diagnostics Ltd.**		**Transasia Bio-medicals Ltd.**		**Bio-Rad Laboratories.**		**Biomerieux.**	
**(Confirmed Positive = 40 Confirmed Negative = 6 0)**	**for each kit**		**Assay result**		**Assay result**		**Assay result**		**Assay result**		**Assay result**	
			**Pos**	**Neg**	**Pos**	**Neg**	**Pos**	**Neg**	**Pos**	**Neg**	**Pos**	**Neg**
	Lot:1	Pos	40	00	40	01	40	00	40	02	40	02
		Neg	00	60	00	59	00	60	00	58	00	58
	Lot:2	Pos	40	01	40	00	40	00	40	02	40	02
		Neg	00	59	00	60	00	60	00	58	00	58
	Lot:3	Pos	40	00	40	00	40	00	40	01	40	01
		Neg	00	0	00	60	00	60	00	59	00	59
	Lot:4	Pos	40	01	40	00	40	00	40	01	40	03
		Neg	00	59	00	60	00	60	00	59	00	57
	Lot:5	Pos	40	00	40	01	40	01	40	02	40	01
		Neg	00	60	00	59	00	59	00	58	00	59

### Results

None of the ELISA kits evaluated was able to identify all the panel members correctly by all the batches (Table 2). But all the kits were found to be 100% sensitive in all the batches. Variation in batches of all the kits was evident in terms of specificity, PPV and efficiency (Table 3). ERBA LISA HIV 1 + 2 provided correct results in 4 batches by identifying all panel samples correctly with 99.8% efficiency. Microlisa HIV and Enzaids HIV 1 + 2 both performed equally by correct result only in 3 batches with 99.6% efficiency. The 4^th ^generation kits, Genscreen and Vironostika, showed the false positivity rates higher than the 3^rd^ generations, but the variations were not statistically significant in terms of specificity (*χ*2 = 0.0683, df = 4, p > 0.05), PPV (*χ*2 = 0.1253, df = 4, p > 0.05) and efficiency (*χ*2 = 0.0230, df = 4, p > 0.05) of all batches of all the ELISA kits. The performance of the kits evaluated using seroconversion panel revealed that all the 3^rd^ generation kits showed equal sensitivity by detecting HIV positivity. In contrast, the 4^th^ generation kits, Genscreen (detected panel member 5, day 16) and Vironostika (detected member 7, day 29) were significantly more sensitive and were able to detect HIV positivity 35 and 22 days earlier respectively than the 3^rd^ generation ELISA kits (Table 4).

**Table 3:** Performance characteristic of HIV ELISA kits used for comparative evaluation

**Kit Name & Company**	**Lot**	**Kit Performance**									
		**Sensitivity**		**Specificity**		**PPV**		**NPV**		**Efficiency**	
		**(%)**	**%Mean (95% CI)**	**(%)**	**%Mean (95% CI)**	**(%)**	**%Mean (95% CI)**	**(%)**	**%Mean (95% CI)**	**(%)**	**%Mean (95% CI)**
Microlisa HIV Company: J. Mitra & Co. Pvt. Ltd.	1	100	100	100	99.3	100	99.0	100	100	100	99.6
			(100-100)		(98.2-100.3)		(97.5-100.4)		(100-100)		(98.9-100.2)
	2	100		98.3		97.6		100		99.0	
	3	100		100		100		100		100	
	4	100		98.3		97.6		100		99.0	
	5	100		100		100		100		100	
Enzaids HIV 1 + 2 ELISA Company: SPAN Diagnostics Ltd.	1	100	100	98.3	99.3	97.6	99.0	100	100	99.0	99.6
	2	100	(100-100)	100	(98.2-100.3)	100	(97.5-100.4)	100	(100-100)	100	(98.9-100.2)
	3	100		100		100		100		100	
	4	100		100		100		100		100	
	5	100		98.3		97.6		100		99.0	
ERBA LISA HIV 1 + 2 Company: Transasia Bio-medicals Ltd.	1	100	100	100	99.6	100	99.5	100	100	100	99.8
	2	100	(100-100)	100	(98.7-100.4)	100	(98.3-100.6)	100	(100-100)	100	(99.3-100.2)
	3	100		100		100		100		100	
	4	100		100		100		100		100	
	5	100		98.3		97.6		100		99.0	
Genscreen Plus HIV Ag-Ab ELISA Company: Bio-Rad Laboratories., U.S.A	1	100	100	96.6	97.2	95.4	96.2	100	100	98.0	98.4
	2	100	(100-100)	96.6	(96.1-98.2)	95.4	(94.8-97.5)	100	(100-100)	98.0	(97.7-99.0)
	3	100		98.3		97.6		100		99.0	
	4	100		98.3		97.6		100		99.0	
	5	100		96.6		95.4		100		98.0	
Vironostika HIV Ag/Ab Company: Biomerieux SA. France	1	100	100	96.6	96.9	95.4	95.8	100	100	98.0	98.2
	2	100	(100-100)	96.6	(95.3-98.4)	95.4	(93.8-97.7)	100	(100-100)	98.0	(97.2-99.1)
	3	100		98.3		97.6		100		99.0	
	4	100		95.0		93.4		100		97.0	
	5	100		98.3		97.6		100		99.0	

CI: Confidence Interval.

**Table 4:** Performance of HIV kits with Seroconversion Panel Sera (Lot# RP-018)

**Panel member**	**Bleed day**	**J Mitra & Co. Pvt. Ltd**	**Span Diag. Ltd.**	**Transasia Bio-Medicals Ltd.**	**Bio-Rad**	**Biomerieux**
		**MICROLISA HIV**	**ENZAIDS HIV 1 + 2**	**ERBALISA**	**GENSCREEN**	**VIRONOSTICA**
		**(S/Co)**	**(S/Co)**	**(S/Co)**	**(S/Co)**	**S/Co)**
1	0	0.022	0.235	0.258	0.224	0.238
2	3	0.007	0.162	0.183	0.221	0.15
3	9	0.007	0.177	0.19	0.238	0.112
4	13	0.015	0.148	0.171	0.238	0.254
5	16	0.015	0.162	0.167	2.425	0.233
6	20	0.007	0.162	0.171	2.888	0.258
7	29	0.65	0.877	0.65	3.291	2.002
8	51	6.723	4.401	3.806	9.396	2.265
9	56	7.511	2.523	2.665	12.724	2.436
10	58	7.285	3.65	4.182	14.224	2.967
11	63	5.226	5.549	4.601	16.388	2.95
12	65	7.635	5.069	5.247	17.993	3.245
13	70	6.211	5.339	5.277	19.828	3.026
14	72	6.073	6.419	5.787	20.91	4.513
15	77	6.825	3.552	3.76	21.045	4.189
16	79	7.182	4.639	4.95	22.425	4.472
17	84	7.328	4.177	4.563	22.993	5.386
18	86	7.81	3.953	4.19	22.037	5.729
19	91	5.401	3.639	4.224	23.187	4.72
20	93	7.606	3.964	4.285	23.575	5.604
21	98	7.372	5.141	5.635	23.56	5.976
22	100	6.467	3.469	3.798	24.007	5.988
23	112	7.562	4.372	4.688	29.881	6.254
24	114	7.993	3.776	4.213	24.91	6.537
25	133	8.825	7.415	7.988	24.858	6.625

S/Co = Sample/cut of.

### Discussion

ELISA is the type of test most commonly used for detection of HIV particularly for large numbers of specimens [2]. But the discordance between the results of different ELISA kits as well as in different lots of the same kit (particularly false positive rates), as evident in this study, highlights an important problem of potentially causing stress to falsely-positive individuals and may also lead to additional expenses [13,14]. Hence, evaluation of diagnostic ELISA kits gains importance for ensuring the availability of suitable kits with better performance in terms of recommended sensitivity and specificity [11], as in case of blood bank testing where a high degree of sensitivity is also recommended for choosing the testing kit [11]. Better performance, comparatively, was observed for ERBALISA kits with 100% efficiency in 4 out of 5 batches (mean efficiency 99.8%). Microlisa and Enzaids each showed mean efficiency of 99.6%. The performance of Genscreen and Vironostika was compromised in terms of specificity, PPV and efficiency as these kits give few false positive results. The PPV as estimated based on the composition of panel sera will change according to the prevalence in the targeted population to be tested [9]. Thus, in an Indian scenario with 0.27% HIV prevalence [15], the estimated PPV of the HIV ELISA kits would be 40.30% for both J.Mitra & Co. Pvt. Ltd. and Span Diagnostics Ltd., 57.40% for Transasia Bio-Medicals Ltd. and 14.40% for Bio-Rad Laboratories. The performance of all five kits in terms of NPV favors their use as a primary screening assay for HIV infection. Unique combination of simultaneous Ag-Ab assay gives better performance in a seroconversion panel as well as reducing the testing window period by 3.82 days on average [6]. In this study, Genscreen and Vironostika, both 4^th^ generation assays, outperformed the 3^rd^ generation ELISA by reducing the window by 35 and 22 days respectively in the seroconversion panel. Genscreen showed better performance and identified early seroconversion than Vironostika in another study [16]. The 3^rd^ generation kits demonstrated efficiency ranging from 98.6 to 99.8%. Though the efficiency of the 4^th^ generation assays is lower, the high sensitivity demonstrated by the kits may favor them for HIV screening purposes, because of their early detection by about 3 weeks post infection. Although lot to lot variation is evident in the study it is not statistically significant (p > 0.05), but kits with specificity level <98% are not recommended for diagnosis of HIV infection in India according to the national guideline [11].

The panel size is small with 100 members and one seroconversion panel only. Kit evaluation with small panel size may be valuable where studies are limited [17]. Use of a range of seroconversion panels is essential to test the biological differences in the timing of appearance of different antibodies to specific antigens in the host response to the various HIV antigens in a range of individuals. A more robust assessment requires the testing of, for example, 10 different seroconversion panels [18,19].

### Conclusion

With 100% sensitivity, both the 3^rd^ and 4^th^ generation commercial ELISA kits seem are appropriate as screening assay for detection of HIV infection. Earlier detection post HIV infection favors the use of 4^th^ generation kits for ensuring HIV free blood for transfusion. The lot to lot variation in terms of specificity warrants batch pre-acceptance testing of all new lots or batches of commercially available ELISA kits in India to ensure that new batches perform as well as previous ones.

## Abbreviations

AIDS: Acquired Immuno Deficiency Syndrome; CDSCO: Central Drug Standard Control Organization; ELISA: Enzyme Linked Immunosorbent Assay; HIV: Human Immunodeficiency Virus; NPV: Negative Predictive Value; PPV: Positive Predictive Value; USFDA: United States Food and Drug Administration.

## Competing interests

The authors declare that they have no competing interests.

## Authors’ contributions

All the authors conceived the study, SN, SM and SCB carried out the laboratory work, MKS analyzed and interpreted the data, SM performed the statistical analysis and all the authors contributed in drafting, and critically reviewed and approved the final manuscript.
